# 

**Published:** 1904-05-21

**Authors:** 


### NOTICE TO CORRESPONDENTS.

All MSS., Letters, Books for Review, and other matters intended for the Editor should be addressed The Editor, "The Hospital," The Hospital Buildings, 28 & 29 Southampton Street, Strand, W.C.

The Editor cannot undertake to return rejected MS., even when accompanied by stamped directed envelope.

**Notice to Advertisers and Subscribers.**

All Advertisements, Orders for Copies of The Hospital, and Business Communications should be addressed to **THE MANAGER (Not to the Editor),** The Hospital, 28 & 29 Southampton Street, Strand, London, W.C.

The Cost of Subscription, post free, is as follows:—Per week, 3½d.; per quarter, 3s. 3d.; per half-year, 6s. 6d.; per annum, 13s. Foreign and Colonial:—Per annum, 19s. 6d., payable, in all cases, in advance.

**NOTICE.—Vol. XXXV. of "The Hospital" (October 1903 to March 1904), in handsome cloth binding, is now on sale at the office of this Journal, price 10s. 6d. Cases for binding the Numbers contained in this Volume 2s. Index to Vol. XXXV., 3½d., post free.**

**The Monthly part for May is now ready. Price 1s., by post, 1s. 3d.**

### BOOKS RECEIVED.

T. Fisher Unwin.

"The Kingdom of Twilight." By Forrest Reid.

Baillière, Tindall and Cox.

"Organic Nervous Diseases." By M. A. Starr.

"Lectures on Clinical Psychiatry." By Dr. E. Kraepelin.

Longmans, Green and Co.

"The Meaning of a Modern Hospital." By W. Bruce Cla[illegible] M.A.

Sidney Appleton.

"The Care and Feeding of Children." By L. Emmett H[illegible] M.D.

C. F. Dowsett.

"Buy English Acres." By C. F. Dowsett.

### Medical Appointments.

**J. R. Armstrong**, M.D., District Medical Officer of the Pontypridd Union. **J. J. Buchan**, M.B., B.Ch.Glasg., D.P.H. Camb., Medical Officer of Health for St. Helen's. **L. Erasmus Ellis**, M.D.Brux., M.R.C.S.Eng., L.R.C.P.Lond., L.S.A.Lond., Clinical Assistant to the Hospital for Consumption and Diseases of the Chest, Brompton, S.W. **H. Morley Fletcher**, M.D.Cantab., F.R.C.P.Lond., Assistant Physician to St. Bartholomew's Hospital. **John Hunter**, M.B., Ch.B.Aberd., House Surgeon to the Aberdeen Royal Infirmary. **Alex. Hutchison**, M.A., M.B., Ch.B.Aberd., Assistant Medical Officer, Kingseat Asylum, Aberdeen Lunacy Board. **W. Howe Lee**, M.R.C.S., L.R.C.P., Medical Attendant, Morton District Hospital under North Derbyshire Hospital Committee. **James McCash**, M.B., C.M.Glasg., House Surgeon to the Royal Eye Hospital, Southwark. **C. de Z. Marshall**, M.R.C.S., L.R.C.P., District Medical Officer of the Tiverton Union. **James Robertson**, M.B., Ch.B.Aberd., House Physician to the Aberdeen Royal Infirmary. **G. B. Sleigh**, M.B., Ch.B.Aberd., Resident Medical Officer to the Royal Aberdeen Hospital for Sick Children. **Enid M. Smith**, M.B., B.S.Lond., Senior Resident Surgeon to the Victoria Hospital, Hull. **Cecil B. F. Tivy**, M.B., B.Ch., B.A.O.R.U.I., Assistant House Surgeon to the Staffordshire General Infirmary, Stafford. **Harold Walker**, M.A., M.B., B.C.Cantab., M.R.C.S., L.R.C.P.Lond., Honorary Surgeon to the North Ormesby Hospital, Middlesbrough. **R. M. Williams**, M.B., C.M.Edin., Certifying Factory Surgeon for the Menai Bridge District, County Anglesey. **R. F. Yencken**, L.R.C.P.&S.Edin., L.F.P.S.Glasg., District Medical Officer of the Bridport Union.

Erratum.—Dr. **J. Wilson Adam** has been appointed Medical Officer to a section of the Aberdeen Town Council employés, and not M.O.H. as stated in our last issue.

**"The Hospital" Institutional Library.**

"Hospitals and Asylums of the World." 4 Vols., with a Portfolio of Plans, complete £12 12s.

"Burdett's Hospitals and Charities, 1904." 5s. net.

"Burdett's Official Nursing Directory." 3s. net.

"Cottage Hospitals, General, Fever, and Convalescent." 10s. 6d.

"Uniform System of Accounts for Hospitals and Public Institutions." New Edition. 4s. net.

"Hospital Expenditure: The Commissariat." 2s. 6d.

"A Legal Handbook for the Use of Hospital Authorities." 2s. 6d.

"The Cottage Hospital Case Book or Register of Patients." 10s. and 12s. 6d.

All these are published by the Scientific Press, Ltd., and may be obtained through any bookseller, or direct from the publishers 28 and 29 Southampton Street, Strand, London, W.C.

### Medical Appointments.

House Surgeon required at once for The Anti-Vivisection Hospital, Battersea. Testimonial to the Secretary. (1848)

### Important Nursing Text-Books.

**A COMPLETE HANDBOOK of MIDWIFERY.**
By J. K. Watson, M.D.Edin., Author of "A Handbook for Nurses."
Crown 8vo. over 350 pp., profusely illustrated with 143 Diagrams.
**6s.** net, **6s. 4d.** post free.

**A HANDBOOK FOR NURSES.**
By J. K. Watson, M.D., M.B., C.M.
New and Revised Edition. Crown 8vo. profusely illustrated, cloth gilt.
**5s.** net, **5s. 4d.** post free.

**NURSING: Its Theory and Practice.**
Being a complete Text-book of Medical, Surgical, and Monthly Nursing.
By Percy G. Lewis, M.D., M.R.C.S., L.S.A.
New Edition, Enlarged and Revised (17th thousand), profusely illustrated with over 100 new Cuts. Crown 8vo. cloth gilt.
**3s. 6d.** post free.

**NURSING IN DISEASES OF THE THROAT, NOSE AND EAR.**
By P. MacLeod Yearsley, F.R.C.S.Eng., M.R.C.S., L.R.C.P.Lond.
Demy 8vo. illustrated, cloth.
**2s. 6d.** post free.

**OPHTHALMIC NURSING.**
By Sydney Stephenson, M.B., F.R.C.S.E.
New and Revised Edition. Crown 8vo. profusely illustrated with Original Drawings, list of Instruments required, and a Glossary of Terms, cloth.
**3s. 6d.** net, **3s. 10d.** post free.

**ART OF MASSAGE.**
By A. Creighton Hale.
Second Edition. Demy 8vo. 150 pp., profusely illustrated with over 70 special Cuts, cloth gilt. **6s.** post free.

**THE HUMAN BODY.**
Its Personal Hygiene and Practical Physiology.
By B. P. Colton, Professor of Natural Science, Illinois University.
Crown 8vo. profusely illustrated, cloth gilt.
**5s.** post free.

**SURGICAL WARD-WORK AND NURSING.**

By Alexander Miles, M.D.Edin., C.M., F.R.C.S E.

Second Edition. Enlarged and entirely Revised. Demy 8vo. copiously illustrated, cloth gilt.

**3s. 6d.**, **3s. 10d.** post free.

**ELEMENTARY ANATOMY AND SURGERY FOR NURSES.**
By William McAdam Eccles, M.S.Lond., M.B., F.R.C.S., L.R.C.P.
Crown 8vo. profusely illustrated, cloth.
**2s. 6d.** post free.

**ELEMENTARY PHYSIOLOGY FOR NURSES.**
By C. F. Marshall, M.D., B.Sc., F.R.C.S.
Crown 8vo. illustrated, cloth. **2s.** post free.

**PRACTICAL GUIDE TO SURGICAL BANDAGING AND DRESSINGS.**
By Wm. Johnson Smith, F.R.C.S.
Demy 16mo. profusely illustrated (suitable for apron pocket), cloth. **2s.** post free.

**THE EXAMINATION OF THE URINE.**

By J. K. Watson, M.D., M.B., C.M., Author of "A Handbook for Nurses."

Demy 16mo. cloth boards.

**1s.** net, **1s. 1d.** post free.

**OUTLINES OF INSANITY.**
A Popular Treatise on the Salient Features of Insanity.
By Francis H. Walmsley, M.D.
Demy 8vo. cloth extra. **3s. 6d.** post free.
Popular Edition, stitched. **1s. 6d.** post free.

**MEDICAL GYMNASTICS, Including the Schott (Nauheim) Movements.**
Being a Text-book of Massage and Mechanical Therapeutics Generally.
By Axel V. Grafstrom, B.Sc., M D.
Crown 8vo. illustrated, cloth. **2s. 6d.**

**IMPORTANT.**

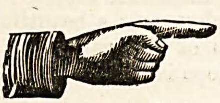

NURSES attending the Medical, Surgical, and Hygienic Exhibition to be held at Queen's Hall, Langham Place, W., next week, should pay a visit to the Scientific Press Stall, No. 58 Avenue E, where the above Text-Books can be seen besides many other useful and up-to-date Nursing Manuals.

